# Missouri Dental College

**Published:** 1888-07

**Authors:** 


### Missouri Dental College.

The twenty-third annual commencement of the Missouri Dental College was held, in connection with that of the St. Louis Medical College, at Memorial Hall, St. Louis, Mo., on Thursday, March 8, 1888.

The degree of D.D.S. was conferred on the following graduates of the Dental Class, by Professor H. H. Mudd, M.D., Dean :

| NAME. | RESIDENCE. | NAME. | RESIDENCE. |
|---|---|---|---|
| William S. Cady | Kansas. | John G. Northington | Kansas. |
| John A. Fries | Missouri. | Murray W. Phillips | Missouri. |
| Jesse E. Grosheider | Indian. | Willard A. Roddy | Missouri. |
| John H. Kennerly | Missouri. | Montana T. Smith | Missouri. |
| Robert E. Keirnan jr | Missouri. | Hugo E. Wangelin | Illinois. |
| Clarence W. Knox | Missouri. | Charles H. Williams | Missouri. |
| H. Muetze, Ph. G | Missouri. | | |

